# Hereditary spastic paraparesis type 46 (SPG46): new *GBA2* variants in a large Italian case series and review of the literature

**DOI:** 10.1007/s10048-024-00749-9

**Published:** 2024-02-09

**Authors:** Ettore Cioffi, Gianluca Coppola, Olimpia Musumeci, Salvatore Gallone, Gabriella Silvestri, Salvatore Rossi, Fiorella Piemonte, Jessica D’Amico, Alessandra Tessa, Filippo Maria Santorelli, Carlo Casali

**Affiliations:** 1 Department of Medico-Surgical Sciences and Biotechnologies, University of Rome Sapienza, Latina, Italy; 2 Department of Experimental and Clinical Medicine, University of Messina, Messina, Italy; 3 Department of Neuroscience and Mental Health, Neurologia 1, A.O.U. Città Della Salute E Della Scienza, 10126 Turin, Italy; 4 Dipartimento Di Neuroscienze, Sez. Neurologia, Facoltà Di Medicina E Chirurgia, Università Cattolica del Sacro Cuore, Rome, Italy; 5 Dipartimento Di Neuroscienze, Organi Di Senso E Torace, UOC Neurologia, Fondazione Policlinico Universitario A. Gemelli IRCCS, Rome, Italy; 6 Unit of Muscular and Neurodegenerative Diseases, Children’s Hospital Bambino Gesù, IRCCS, Rome, Italy; 7 IRCCS Stella Maris Foundation, Calambrone, Via Dei Giacinti 2, 56128 Pisa, Italy

**Keywords:** Hereditary spastic paraparesis, Non-lysosomal glucosylceramidase, Literature review

## Abstract

Hereditary spastic paraparesis (HSP) is a group of central nervous system diseases primarily affecting the spinal upper motor neurons, with different inheritance patterns and phenotypes. SPG46 is a rare, early-onset and autosomal recessive HSP, linked to biallelic *GBA2* mutations. About thirty families have been described worldwide, with different phenotypes like complicated HSP, recessive cerebellar ataxia or Marinesco-Sjögren Syndrome. Herein, we report five SPG46 patients harbouring five novel *GBA2* mutations, the largest series described in Italy so far. Probands were enrolled in five different centres and underwent neurological examination, clinical cognitive assessment, column imaging for scoliosis assessment, ophthalmologic examination, brain imaging, GBA2 activity in peripheral blood cells and genetic testing. Their phenotype was consistent with HSP, with notable features like upper gaze palsy and movement disorders. We review demographic, genetic, biochemical and clinical information from all documented cases in the existing literature, focusing on the global distribution of cases, the features of the syndrome, its variable presentation, new potential identifying features and the significance of measuring GBA2 enzyme activity.

## Introduction

Hereditary spastic paraparesis (HSP) represents a group of central nervous system (CNS) diseases that mainly involve the spinal portion of upper motor neurons [1]. Hallmark pathologic alteration in HSPs is diffuse axonal “dying-back” degeneration, most represented in the terminal segments of the longest axons, with possible involvement of dorsal columns [2, 3]. Involvement of the lower motor neurons can also be observed [4]. HSPs have an estimated prevalence ranging from 2 to 4.1 in 10^4^ individuals [5, 6], and they are classified according to inheritance (autosomal dominant, autosomal recessive, X-linked or mitochondrial with maternal trait transmission), to phenotype (“pure” or “complex”) and to onset (early or late) [7, 8]. The pathogenesis of HSP is connected to a wide range of cellular processes, including membrane and axonal transport, modulation of the endoplasmic reticulum membrane, mitochondrial function, DNA repair, autophagy, lipid metabolism and myelination. Additionally, dysfunction in endosome membrane trafficking, oxidative stress and mitochondrial DNA polymorphisms have been implicated [9].

Phenotypically, “pure” HSPs [10] typically manifest with pyramidal signs starting in lower limbs, associated with variable disorders as sphincter dysfunctions and deep sensory loss. On the other hand, “complex” HSPs may display a broader range of neurological manifestations including cerebellar dysfunction, peripheral neuropathy, extrapyramidal features, seizures, deafness, cognitive impairment and psychiatric disorders [1, 5]. Extraneurological manifestations can include cataracts, optic neuropathy, retinitis pigmentosa, facial dysmorphisms, scoliosis, hip dislocation and different foot deformities [1, 11]. Furthermore, CNS neuroimaging reveals characteristic features, often related to a specific subtype: cerebellar atrophy, thin corpus callosum (TCC), white matter abnormalities (WMA), spinal cord atrophy, brain iron accumulation and hydrocephalus [12–14]. Within the group of autosomal recessive hereditary spastic paraplegias (ARHSP), SPG11 is the most common, followed by SPG7, SPG15 and SPG56, whereas SPG46 seems more rare [15–21].

SPG46 is a rare, early onset spastic paraparesis, classified as “complex” [5]. It is inherited in an autosomal recessive manner, and its clinical presentation has appeared strikingly different from other ARHSP since its initial and seminal description [22]. It is associated with biallelic mutations in the *GBA2* gene (locus 9p13.3) encoding for the non-lysosomal glucosylceramidase (GBA2) [20, 23], a ubiquitous enzyme associated with the endoplasmic reticulum and the plasma membrane, which catalyzes conversion of glucosylceramide to glucose and ceramide [24]. The other (non-homologous) enzyme degrading glucosylceramide is the lysosomal acid β-glucosidase (GBA), whose mutations cause Gaucher’s disease and a form of hereditary Parkinson’s disease [25]. Glucosylceramide is the precursor component of gangliosides. Mutations in the *GBA2* lead to changes in enzymatic activity, which can be detected in lymphoblasts and leucocytes of affected subjects [26], but also affect neurons [23, 27], resulting in abnormal increase of glucosylceramide, although the pathogenic mechanism of neurodegeneration is still unclear [24]. *GBA2* mutations seem to lead to a clinical spectrum that encompasses different phenotypes, including HSP, autosomal recessive cerebellar ataxia (ARCA), or a more complex and severe condition like Marinesco-Sjögren Syndrome (MSS) [18, 27, 28]. Clinically, HSP46 may show plethoric signs, both neurological and extraneurological, besides spastic paraparesis, such as: cerebellar dysfunction, peripheral neuropathy, distal amyotrophy, cognitive impairment, scoliosis, cataracts [16, 20, 29], and there is still not a consensus basis about the overall definite phenotype. Furthermore, not all reports contain information about the features thought to be hallmarks, or at least common, in this disease (Table 1). Brain MRI may display WMA, TCC, cerebral, brainstem and cerebellar atrophy. To date, more than 90 genetic types of HSP have been identified [5, 15] (https://neuromuscular.wustl.edu/spinal/fsp.html; *OMIM*), and 62 patients, between isolated cases and families, with SPG46 have been described worldwide. Since its clinical discovery [22], when its genetic mutation was still unknown, and subsequent identification of the causative mutation [16], 23 reports about SPG46 have been published. In over 15 years, different phenotypes have been described, pointing out a phenotypical heterogeneity and reinforcing the concept of “clinical spectrum” of this disease. Herein we report five novel *GBA2* pathogenic variants detected in unrelated SPG46 Italian patients. We also provide a comprehensive review of demographic, genetic, biochemical and clinical data from all SPG46 cases described in the existing literature, discussing about cases’ global distribution, overall phenotype’s characteristics, variable expressivity, possible hallmarks and the importance of GBA2 activity dosage.

**Table 1:** Clinical, demographic, radiological and biochemical details of our patients

		Probands				
	A	B	C	D	E	Mean
*Features*						
Males/females	M	M	M	F	M	4 M/1F
Consanguinity	Yes	No	No	No	Yes	2/5
World area	Morocco	Italy	Italy	Italy	Italy	
Age last examination (y)	36	33	56	21	47	38,6
Onset (y)	10	0	12	2	10	6,8
Progression (y)	26	33	44	20	37	32
Spasticity	Yes	Yes	Yes	Yes	Yes	5/5
Sphincteric symptoms	Yes	Yes	No	Yes	No	3/5
Cerebellar syndrome	Yes	Yes	No	Yes	Yes	4/5
Peripheral neuropathy	Yes	Yes	Yes	No	Yes	4/5
MCI	No	Yes	Yes	Yes	Yes	4/5
Cataract	Yes	Yes	No	Yes	Yes	4/5
Scoliosis	Yes	Yes	No	No	No	2/5
Pes cavus	Yes	No	No	No	No	1/5
Hypogonadism	No	No	No	-	No	0/4
*Movement disorders*	No	No	Yes	No	No	1/5
Head tremor	No	No	No	No	No	0/5
Cervical/facial dystonia	No	No	No	No	No	0/5
Facial myokimias	No	No	No	No	No	0/5
Limb dystonia	No	No	Yes	No	No	1/5
Limb tremor	No	No	Yes	No	No	1/5
*Other symptoms/signs*	UGP	No	UGP	No	No	2/5
SPRS	23	31	NA	11	30	23.75
*Overall phenotype*						
HSP ph	Yes	Yes	Yes	Yes	Yes	5/5
ARCA ph	-	-	-	-	-	0/5
M-S ph	-	-	-	-	-	0/5
*MRI*						5/5
TCC	Yes	No	No	Yes	No	2/5
Cerebellar atrophy	Yes	No	No	No	No	1/5
Brainstem atrophy	Yes	No	No	Yes	No	2/5
Cerebral atrophy	No	No	No	No	No	0/5
WMA	Yes	No	Yes	Yes	Yes	4/5
*GBA2 activity probands*	0.28 nmol/mg	0.01 nmol/mg	Pending	Pending	NA	0.145 nmol/mg

## Methods

### Patients

This multicentric case series study was performed in accordance with the Declaration of Helsinki and its later amendments. Written informed consent and ethical approval (CE Lazio) were obtained. In a single laboratory, we tested about 735 patients with clinical evidence of HSP without genetic diagnosis, using a multigene targeted resequencing panel and investigating the coding exons and flanking introns of the genes known to be associated with HSPs [5, 15, 30]. From September to November 2021, five patients harbouring biallelic *GBA2* mutations (four men; one woman) were identified and recruited in five Italian neurology centres (University of Rome Sapienza, Policlinico Universitario A. Gemelli IRCCS in Rome, “Città della Salute e della Scienza” in Torino, IRCCS Stella Maris Foundation in Pisa, University of Messina). These patients were enrolled in the study and underwent further investigation and analysis. Family and clinical history were collected. All patients underwent neurological examination, clinical cognitive assessment through Mini Mental State Examination (MMSE) [31], spinal radiological study (for scoliosis assessment), ophthalmologic examination (for cataract assessment), brain MRI (Table 1).

### GBA2 activity measurement

**Fig. 1:**
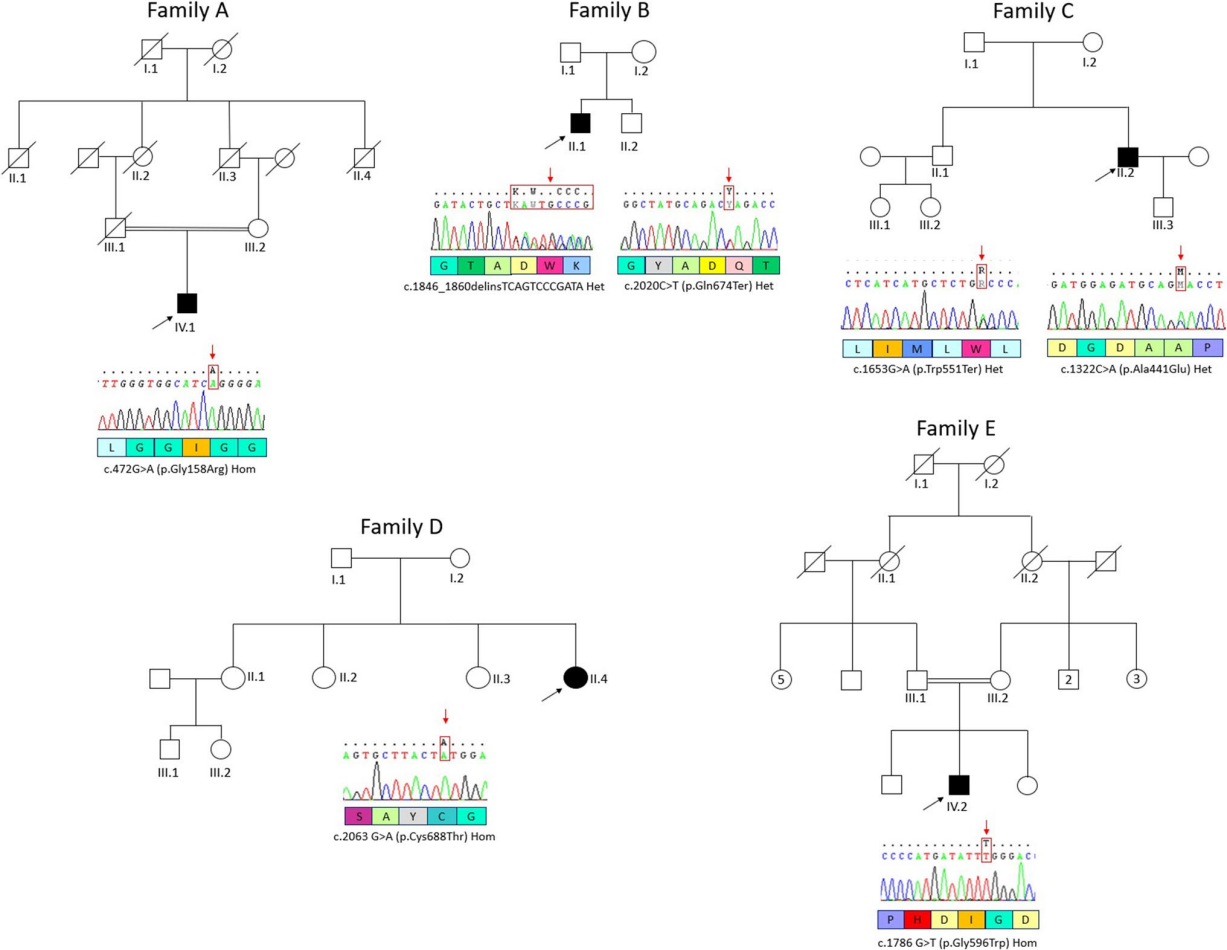
Families’ pedigree; family’s letter indicates each proband; arrows indicate the probands; chromatograms and mutations on bottom of each pedigree

Two of five index cases (Table 1; Fig. 1, proband A and B) underwent GBA2 enzyme dosage [32]. Leucocytes were isolated from ~ 6-mL EDTA blood as previously described [33], and kept at − 20 °C for 1 day before being thawed and maintained on ice until the start of the enzyme reaction. For the isolation of leucocytes, 10% dextran was added. Erythrocytes were allowed to sediment at room temperature for 45 min and the upper phase centrifuged at 1125 × g for 10 min. The pellet was washed with 0.9% NaCl and stored at − 20 °C until the analysis. Protein content was determined by the BCA protein assay (Pierce, Rockford, USA). Leucocytes were diluted in 100 µL of deionized water and sonicated three times for 2 s by a Vibra Cell Sonicator (Sonics-Materials Inc., VCX130, Newton, Connecticut, USA). Stock solutions of substrate and inhibitor were prepared as follows: Substrate: 4-methylumbelliferone-β-glucopyranoside 10 mM (Glycosinth, Warrington, UK) in citric acid 200 mM (Sigma-Aldrich, St. Louis, MO, USA) and disodium hydrogen phosphate 100 mM (Merck, VWR International), pH 5.2. Inhibitor: conduritol β epoxide (CBE) (0.26 M) (Sigma-Aldrich, St. Louis, MO, USA)). Ten microliters of sample (30-µg protein) with and without 10 µL of inhibitor were made up to 50 µl with deionized water and pre-incubated for 1 min at 37 °C. The assay was initiated by adding 100 µl of substrate. Duplicates of each sample with and without inhibitor, as well as duplicates blanks (10 µl of deionized water and 100 µl of substrate) were incubated in 2-mL eppendorf tubes for 120 min at 37 °C in a water bath with orbital shaker. The reaction was stopped with (0.5 M) NaHCO_3_/(0.5 M) Na_2_CO_3_ pH 10.3 and fluorescence of samples, blanks and standard solution (4-methyl umbelliferone 0.5 mM, diluted in (0.5 M) NaHCO_3_/(0.5 M) Na_2_CO_3_ pH 10.3) were measured within 1 h (shielded from light) in a spectrofluorometer ( FL6500 Perkin Elmer, Waltham, MA, USA) using an excitation wavelength of 365 nm and an emission wavelength of 448 nm. The activity of GBA2 was calculated according to the standard curve and expressed as nmol/mg proteins.

### Molecular and database search

DNA extraction was carried out using peripheral blood lymphocytes obtained from the patients. Subsequently, Next Generation Sequencing (NGS) analysis was performed using an amplicon based customized NGS panel (Illumina TrueSeq Custom Amplicon, TSCA) including more than 200 genes involved in HSP pathogenesis. Literature was reviewed using PubMed and Google Scholar, and findings were collected in Tables 3, 4 and 5. Excel was employed to create map graphics. Search for variants of *GBA2* was done using population databases (dbSNP, 1000genome, EVS) and local databases, and their pathogenicity were assessed according to the American College of Medical Genetics and Genomics (ACMG) guidelines [34].

## Results

### Cases clinical reports

**Fig. 2:**
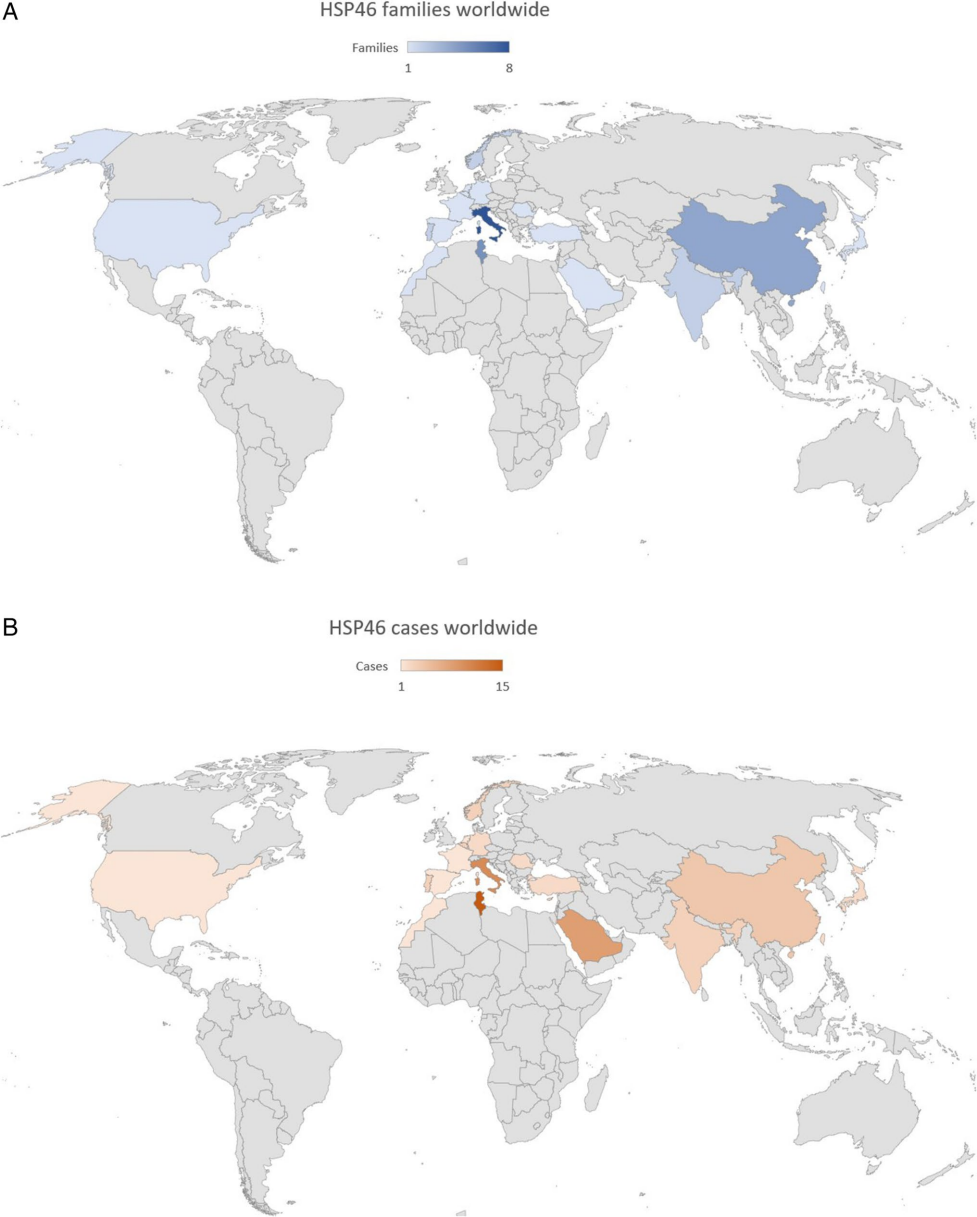
**A** Families’ distribution per country; the colour’s intensity shows the prevalence. **B** Cases’ distribution per country; the colour’s intensity shows the prevalence

Patients’ clinical, imaging and laboratory features are summarized in Table 1. Pedigrees are shown in Fig. 1. Consanguinity of the patients’ parents was identified in two out of five cases (Fig. 1, families A and E). All families, except for one, had Italian descent (Family A came from Morocco—Table 2, Figs.1 and 2). Except for one proband, all individuals had early disease onset (between 6 and 7 years—mean 6.8 years). In one case (proband B) we found congenital onset. The overall initial manifestation was spasticity in the lower limbs (5/5), subsequently followed by various neurological and extraneurological manifestations which, with some degree of variability, enriched the clinical picture (Table 1): cerebellar syndrome (4/5), sphincteric symptoms (like urge incontinence, 3/5), intellectual disability (MCI, 4/5), peripheral neuropathy (4/5), movement disorders like UL tremor and dystonia (1/5), bilateral cataracts (4/5), scoliosis (2/5) and pes cavus (1/5). Mean SPRS score was 23.75 (NA in one case). At the time of last neurological examination, the mean age of the patients was 38.6 years. In two patients, we found ocular movement disorder (upper gaze palsy (UGP)). MMSE assessment was administered, mainly resulting in MCI (except proband A). No one of the male patients had hypogonadism. They all underwent spine RX and brain MRI: the most frequent sign was WMA (4/5), followed by TCC (2/5). Two patients had skeletal deformities, comprising scoliosis and/or pes cavus. Disease course was slowly progressive (mean 32 years at the time of last examination).

**Table 2:** *About the report from Saudi Arabia, only the proband’s results were published; about the other seven members, it has only been referred they had spastic paraparesis and cerebellar ataxia, but the report lacks any additional data from them

Countries (sort by time of report)	Families	Cases
Tunisia	5	15
Belgium	1	3
Turkey	1	2
Portugal	2	3
Cyprus	1	3
Italy	8	10
Romania	1	2
Netherlands	1	1
China	4	4
Norway	2	3
France	1	1
Saudi Arabia*	1	8
Japan	1	2
Germany	1	2
India	2	3
Taiwan	1	2
USA	1	1
Spain	1	1
Morocco	1	1

### Molecular findings

Gene testing identified 7 *GBA2* variants, four of which were compound heterozygous (Fig. 1). The c.472G > A (p.Gly158Arg) and c.2063 G > A (p.Cys688Thr) pathogenic variants have already been reported [35, 36]. The other five variants, i.e. the homozygous c.1786 G > T (p.Gly596Trp) and the four compound heterozygous (c.1846_1860delinsTCAGTCCCGATA + c.2020C > T (p.Gln674*) and c.1653G > A (p.Trp551*) + c.1322C > A (p.Ala441Glu), were absent in our in-house databases as well as in population databases (dbSNP, 1000genome, gnomAD) and were classified as likely pathogenic according to the ACMG guidelines. Missense variants were indicated as “probably damaging” and “damaging” by two in silico predictors (PolyPhen-2 and SIFT).

### Biochemical findings

Two patients underwent analysis of enzymatic activity, while it was not available for three. We determined the GBA2 activity in leucocytes as the beta-glucosidase activity that is resistant to 2.5 mM conduritol B epoxide. This activity was much reduced in proband A and B (Table 1; 0.28 nmol/mg and 0 0.01 nmol/mg, respectively) by comparison with the mean values of 5 controls (3.9; reference range: 2.5 to 5.3 nmol/mg). We acknowledge that this method of measuring GBA2 activity may lead to underestimating GBA2 activity [37].

## Discussion

We present five previously unreported Italian patients with SPG46. These patients were found to have five novel *GBA2* variants, all of which were determined to be pathogenic based on in silico predictors. This report represents the 24th documented study on the disease (Table 3, 4 and 5). Thus far, a total of 67 cases (30 men, 34 women, sex not specified in three) from 36 families have been described worldwide (Tables 2 and 3; Fig. 2) [16–21, 27, 29, 35, 36, 38–50] since the seminal description of Boukhris et al. in 2008 [22]. Patients with *GBA2* pathogenic variants have been described in Tunisia, Belgium, Turkey, Portugal, Cyprus, Italy, Romania, Netherlands, China, Norway, France, Saudi Arabia, Japan, Germany, India, Taiwan, USA and Spain [16–21, 27, 29, 35, 36, 38–50] (Fig. 2 and Table 2). Based on the worldwide distribution, the prevalence of the disease seems to be higher in the Mediterranean area (Fig. 2). It ought to be considered the prevalence in each country where the highest number of cases has been reported (Table 2). In Tunisia, for instance, there have been 15 cases reported, with a prevalence of approximately 15 in 1.2 × 10^7^ individuals. Similarly, in Italy, there have been 10 reported cases in approximately 5.9 × 10^7^ individuals. Saudi Arabia has reported 8 cases, resulting in a prevalence of about 8 in 3.6 × 10^7^ individuals. Lastly, in China, 4 cases have been documented, indicating a prevalence of around 4 in 140 × 10^7^ individuals. Considering these prevalence rates, the Mediterranean area exhibits the highest concentration of SPG46 cases due to its smaller population (Fig. 2). For instance, this is particularly notable when comparing it with countries like the USA, which has a significantly larger population (approximately 33 × 10^7^ inhabitants) but a minimal prevalence of the disease, with only one reported case so far [35]. Since ARHSPs are more common in countries with a higher rate of consanguinity, this may provide an explanation for the higher number of reported cases in these areas. A thorough demographical, clinical, radiological and biochemical comparison was conducted, examining the features of our cases in relationship with the available literature (Tables 1, 3, 4 and 5). Two out of five patients included in this study belonged to consanguineous families, where the parents were found to be relatives (Fig. 1; Table 1). Consanguinity is commonly observed in recessive diseases, and SPG46 makes no exception. Among the available reports in literature with this information (12 out of 23), consanguinity was investigated in 19 families (out of 31) and was found in 14 of them (73.68%) (Table 3). This highlights the prevalence of consanguineous marriages within the context of SPG46 and underscores the significance of genetic factors in the disease’s inheritance patterns. In all cases reported so far, the presence of both spastic paraparesis and cerebellar syndrome has been consistently observed (Table 3). In our study, all patients had early onset (6.8 year) and slow progression over time. Remarkably, one case had congenital onset (Table 1; proband B), and one had the longest progression so far (Table 1; proband C—44 years of disease); it is the second reported with such disease duration [47, 50]. Additional clinical features, commonly regarded as characteristic signs of this rare HSP [16–20, 22] such as neuropathy, MCI, bilateral cataracts, scoliosis, pes cavus and hypogonadism are observed with varying prevalence among the SPG46 population (Table 3). MCI is a common feature (Tables 1 and 3), but it may show very lately [18]. About half of the cases described so far show MCI, but its prevalence may turn out to be higher, due to later onset, as in our proband C (Table 1).

**Table 3:** Details about clinical and demographical findings in all HSP46 cases so far

Reports with/without data (or NA)			13/11	20/4	10/14	24/0	24/0	17/7	15/9	18/6	12/12	13/11	11/13
	Cases	Families	Consanguinity (families)	Onset (y)	Progression (y)	Spasticity	Cerebellar syndrome	Peripheral neuropathy	MCI	Cataract	Scoliosis	Foot abnormalities	Hypogonadism (males)
Boukhris A et al. (2010)	5	1	Yes	4,4	NR	5	5	5	5	5	No	5	NR
Hammer HB et al. (2013)	10	4	4 yes	8,4	26	10	10	10	1	NR	3	3	No
Martin E et al. (2013)	11*	4*	3 yes, 1 no	8,5	23	11*	11*	5	11*	11*	No	5*	2
Votsi C et al. (2014)	3	1	Yes	15,7	41	3	3	3	3	1	NR	3	NR
Citterio A et al. (2014)	3	1	Yes	10	25	3	3	No	3	No	No	2 (flat foot)	No
Kancheva D et al. (2016)	2	1	NR	NR	14	2	2	2	NR	NR	NR	NR	NR
van de Warrenburg BP et al. (2016)	1	1	NR	3	NR	1	1	1	1	1	NR	NR	NR
Yang YJ et al. (2016)	1	1	Yes	8	NR	1	1	NR	1 (dementia)	NR	NR	NR	NR
Haugarvoll K et al. (2017)	3	2	2 no	6	NR	3	3	3	3	3	1	1	1
Morais S et al. (2017)	2	1	NR	10	NR	2	2	2	NR	NR	NR	NR	NR
Coutelier M et al. (2018)	1	1	NR	NR	NR	1	1	1	NR	1	NR	NR	NR
Coarelli et al. (2018)	1	1	No	6	NR	1	1	1	1	No	no	1	No
Wei Q et al. (2019)	1	1	NR	22	NR	1	1	1	NR	No	NR	NR	NR
Algahtani H et al. (2019)^*^	8	1	Yes	12 (proband)	NR	8	8	NR	NR	1	No	No	No (woman)
Guan RY et al. (2020)	2	2	NR	NR	NR	2	2	2	2	NR	NR	NR	NR
Spagnoli C et al. (2020)	1	1	NR	NR	NR	1	1	NR	NR	No	No	No	No (woman)
Nakamura-Shindo K et al. (2020)	2	1	Yes	23	NR	2	2	NR	NR	2	No	No	No
Kloth K et al. (2020)	2	1	NR	25	14	2	1	1	2	1	No	No	No (women)
Holla VV et al. (2021)	3	2	2 yes	7	28	3	2	NR	1	No	NR	NR	NR
Gatti M et al. (2021)	1	1	NR	2	15	1	1	1	1	1	1	No	No (woman)
Lan MY et al. (2022)	2	1	NR	20,5	39	2	2	NR	2	No	NR	NR	NR
Gill et al. (2023)	1	1	NR	9	NR	1	1	NR	NR	NR	NR	NR	NR
Cores Bartolomé C et al. (2023)	1	1	No	10	NR	1	1	1	NR	1	NR	1 (+ equinus)	No (woman)
Present report	5	5	2 yes, 3 no	6,8	44	5	5	4	4	4	2	1	No
Total	67	36	16 yes, 8 no	10,9	26,9	67 (100%)	67 (100%)	**43 (87.76%)**	**36 (75%)**	**27 (64.3%)**	**7 (17.5%)**	**17 (38.6%)**	3 (16.7%)

*Yes* present, *No* absent, *NA* not available, *NR* not reported

^*^Cases include the same Tunisian family (5 cases) described by Boukhris et al. in 2008 and 2010; ∗ 7 cases were assessed as spastic-ataxic, but not further examined; among foot abnormalities, only 2 out of 44 cases had flat foot, while other cases had pes cavus; in the last line, in bold characters, there are the features considered as hallmarks

**Table 4:** Details about clinical and demographical findings in all HSP46 cases so far

Reports with/without data (or NA)		17/7						16/8							
	Cases	Movement disorders	*Head tremor*	*Cervical/facial dystonia*	*Facial myokimias*	*Limb dystonia*	*Limb tremor*	Other symptoms/signs	*Upper gaze palsy*	*Hearing loss*	*Eyelid ptosis*	*Developemental delay*	*Delusions*	*Anxiety*	*Learning disorder*
Boukhris A et al. (2010)	5	No						No							
Hammer HB et al. (2013)	10	Yes	3					No							
Martin E et al. (2013)	11*	No						Yes		3					
Votsi C et al. (2014)	3	No						Yes	3	3					
Citterio A et al. (2014)	3	NR						Yes			3				
Kancheva D et al. (2016)	2	NR						NR							
van de Warrenburg BP et al. (2016)	1	NR						NR							
Yang YJ et al. (2016)	1	Yes				1		Yes	1						
Haugarvoll K et al. (2017)	3	Yes	2	1 (+ Athetosis)			2	No							
Morais S et al. (2017)	2	Yes				2		NR							
Coutelier M et al. (2018)	1	NR						NR							
Coarelli et al. (2018)	1	Yes					1	No							
Wei Q et al. (2019)	1	No						Yes					1	1	
Algahtani H et al. (2019)^*^	8	Yes	1				1	NR							
Guan RY et al. (2020)	2	NR						NR							
Spagnoli C et al. (2020)	1	No						Yes							1
Nakamura-Shindo K et al. (2020)	2	No						Yes					1		
Kloth K et al. (2020)	2	Yes	1	2		1 (writer cramp)		Yes	1						
Holla VV et al. (2021)	3	Yes	1 (no–no head tremor)	1		2	1	Yes	2			2			
Gatti M et al. (2021)	1	Yes			1		1	No							
Lan MY et al. (2022)	2	NR						NR							
Gill et al. (2023)	1	NR						NR							
Cores Bartolomé C et al. (2023)	1	Yes				1		No							
Present report	5	Yes				1	1	Yes	2						
Total†	67	**17/48 (35.4%)**	**9/48 (18.75%)**	5/48 (10.4%)	1/48 (2%)	**8/48 (16.7%)**	7/48 (14.6%)	**18/48 (37.5%)**	**9/48 (18.8%)**	6/48 (12.5%)	3/48 (6.25%)	2/48 (4.2%)	2/48 (4.2%)	1/48 (2%)	1/48 (2%)

*Yes* present, *No* absent, *NA* not available, *NR* not reported

^*^Cases and families include the same Tunisian family (5 cases) described by Boukhris et al. in 2008 and 2010; ∗ 7 cases were assessed as spastic-ataxic, but not further examined

^†^The second column shows the total number of HSP46 cases, while the third column shows the number of cases in which specific data was found among the total cases examined; in the last line, in bold characters, there are the features we propose to take into consideration in clinical diagnosis

**Table 5:** Details about clinical and demographical findings in all HSP46 cases so far

Reports with/without data (or NA)					19/5							5/19
	Cases	HSP ph	ARCA ph	MSS ph	Brain MRI	Positive MRI	*TCC*	*Cerebellar atrophy*	*Brainstem atrophy*	*Cerebral atrophy*	*WMA*	Patients assayed for GBA2 activity
Boukhris A et al. (2010)	5	x			Yes	4	2	2		1		NA
Hammer HB et al. (2013)	10		x		Yes	1	1					NA
Martin E et al. (2013)	11*	x			Yes	2	2	2				NA
Votsi C et al. (2014)	3		x		Yes	3		3				3(GBA2 activity almost undetectable)^**^
Citterio A et al. (2014)	3		x		NA							NR
Kancheva D et al. (2016)	2	x			NA							NR
van de Warrenburg BP et al. (2016)	1	x			Yes	1	1				1	NR
Yang YJ et al. (2016)	1	x			Yes	1					1	NR
Haugarvoll K et al. (2017)	3			x	1 NA, 2 yes	2	2	2		2		1 (7% residual GBA2 activity)
Morais S et al. (2017)	2	x			NA							NR
Coutelier M et al. (2018)	1	x			NA							NR
Coarelli et al. (2018)	1	x			Yes	1	1	1				NR
Wei Q et al. (2019)	1	x			Yes	1 neg						NR
Algahtani H et al. (2019)^*^	8	x			7 NA, 1 yes	1		1				NR
Guan RY et al. (2020)	2	x			NA							NR
Spagnoli C et al. (2020)	1	x			Yes	1	1					NR
Nakamura-Shindo K et al. (2020)	2	x			1 NA, 1 yes	1	1	1	1			NR
Kloth K et al. (2020)	2	x			1 NA, 1 yes	1			1		1	2 (GBA2 activity severely reduced)
Holla VV et al. (2021)	3	x			1 NA, 2 yes	2	2	2				NR
Gatti M et al. (2021)	1	x			Yes	1 neg						1 (almost indetectable GBA2 activity)
Lan MY et al. (2022)	2	x			Yes	2		2				NR
Gill et al. (2023)	1	x			Yes	1	1					NR
Cores Bartolomé C et al. (2023)	1	x			Yes	1 neg						NR
Present report	5	x			Yes	5	2	0	1	0	4	2
Total†	67				46/67 (68,6%)	29/46 (63%)	16 (35%)	16 (35%)	3 (7%)	3 (7%)	7 (15%)	9/14

*Yes* present, *No* absent, *NA* not available, *NR* not reported

^*^Cases and families include the same Tunisian family (5 cases) described by Boukhris et al. in 2008 and 2010

^**^GBA2 activity dosage done in 2018 on this family. Two-fold increase of glucosylceramide; GBA1 activity was three-fold increased; * 7 cases were assessed as spastic-ataxic, but not further examined

^†^The second column shows the total number of HSP46 cases, while the third column shows the number of cases in which specific data was found among the total cases examined

Movement disorders, like head and upper limbs’ tremor, cranial and upper limbs’ dystonia, can be observed with moderate occurrence, and they appear to be part of the clinical presentation in several described cases (17 out of 55—Table 4). Cervical dystonia has been outlined as the onset symptom in one patient, later evolved into a complex athetotic-dystonic disorder which involved the UL (initially described as “writer’s cramp”); at brain MRI she showed brainstem atrophy, also involving basal ganglia [47]. Facial myokymias were reported too [29]. Other neurological signs and symptoms emerged (Tables 1 and 4). Six cases of hearing loss have been reported. This is a symptom frequently found in mitochondrial diseases [51, 52]. Since a role in mitochondrial fragmentation has been already outlined in *GBA2* mutation [53], we may suppose a similar mechanism in SPG46. Four cases of psychiatric disorders are also described [36, 43, 46]: in one case, the disease onset was represented by delusions [43]. Among the other neurological signs, UGP is the most frequent (19%—Tables 1 and 4) [18, 21, 47, 48]. Interestingly, this phenomenon is frequently observed in Gaucher’s Disease Type 3 (GD3). It is attributed to ceramide accumulation in cerebellar and brainstem areas controlling vertical gaze: floccular lobe, vestibular system, pontine paramedian reticular formation, rostral interstitial nucleus of the medial longitudinal fascicle and motor neurons of the abducens nucleus [54, 55]. GD3 is a neurodegenerative disease caused by pathological accumulation of glucosylceramide in the CNS due to lysosomal GBA dysfunction [56], similarly to what happens in SPG46. GBA and GBA2 do not have the same location or functioning [57]. However, it has been pointed out not only an akin role (i.e. glucosylceramide metabolism), but also an indirect action synergy [58]. Malekkou et al. biochemically characterized the same Cypriot SPG46 family described in 2014 [18], and, besides abolished GBA2 activity, they highlighted a compensatory effect of GBA, since its activity was threefold higher in SPG46 patients compared to controls [26]. Thus, the two enzymes not only share a similar role, but seem also to be related. Since UGP seems to be recurrent in SPG46 (Tables 1 and 4), we hypothesize a similar role of GBA2, resulting in brainstem and cerebellar dysfunction, and thus leading to UGP.

With exception of peripheral neuropathy and cognitive assessment, many reports lack additional clinical data or do not provide negative results (Tables 3, 4 and 5). As shown in Table 3, features such as scoliosis, foot abnormalities and hypogonadism should undergo more comprehensive investigation to determine their status as defining characteristics. This presents a challenge in further delineating the phenotypic profile of SPG46. Furthermore, manifestations like movement disorders (dystonia), ocular movements abnormalities and skeletal deformities might have a higher occurrence (Table 4). In future reports, recognizing and considering these features can aid diagnosis.

In most reports (20 out of 24, which includes our own study—Table 5) patients and families are primarily described with a phenotype consistent with HSP. Only few descriptions classify the disease as ARCA [17–19], and just one report identifies it as MSS [27]. That may often depend on predominant symptoms, or on signs and symptoms at onset. In 2013, Hammer et al. discovered the second group of *GBA2*-mutated patients, and initially diagnosed the condition as autosomal recessive ataxia, as the first presentation involved cerebellar syndrome. However, shortly thereafter, in addition to peripheral neuropathy, significant spasticity emerged, initially in the lower limbs and subsequently extending to the upper limbs, becoming highly pronounced and dominating the overall clinical presentation [19]. Later, Votsi et al. discovered a new *GBA2*-mutated family, in Cyprus, and classified it as ARCA. However, their phenotype description involves a typical HSP onset and progression (with spasticity in lower limbs) [18]. The first Italian description of SPG46, depicted as ARCA, involved three affected individuals from the same family. Still, a predominantly cerebellar phenotype was evident in a single case, while the other members displayed HSP, and significant intrafamilial variability [17]. Curiously, the variant discovered by Hammer et al. (c.2618G > A) [19] is the same of the Saudi SPG46 family from 2019 [44], which was described mainly as a HSP, complicated by cerebellar ataxia. Thus, different phenotypes may arise from same identical mutations, as also evidenced by the intrafamilial variability observed by Citterio et al. [17]. There are cases where the same genotype causes different degrees of phenotypical expression, even in the same family, as seen in diseases like neurofibromatosis, Van der Woude syndrome or holoprosencephaly [59–61]. Differently from incomplete penetrance, in which the expected phenotype manifests or not, this phenomenon is referred to as “variable expressivity”, which quantifies the degree to which a genotype displays its phenotypic expression [62]. Variable expressivity appears to be caused by a range of factors, including common variants, variants in regulatory regions, gene-modifiers, epigenetics, environmental factors and lifestyle [63]. In the two Norwegian families described in 2017, the probands presented at examination with early onset cerebellar ataxia, also with bilateral cataract, mental retardation and late spastic paraparesis [27]. Clinical diagnosis of MSS was made (one of the families was visited and diagnosed in 1977) [64]. MSS is an AR disorder, caused by mutations in *SIL1*, characterized by cerebellar atrophy with ataxia, early-onset cataracts, and it also may include mild to severe intellectual disability, hypogonadism and skeletal abnormalities [65–67]. Its clinical hallmarks are child-onset hypotonia and muscle weakness but not spasticity in lower limbs [68]. The Norwegian patients did not exhibit hypotonia or myopathy during childhood and instead presented with early onset-spastic paraparesis. Therefore, SPG46 may start with diverse symptoms sometimes different from spasticity in lower limbs, like cerebellar ataxia and may seldom show different phenotypes [17–19, 27]. Regardless of different onset or dominant symptoms, the disease is primarily described in most reports as a complex form of ARHSP. Considering the long disease duration (mean 26.9 years—Tables 1 and 3), the overall phenotype gradually manifests as a “typical” complex ARHSP over time, exhibiting distinct features that serve as hallmarks of SPG46 (Tables 1, 3, 4 and 5). At times, the differentiation between cerebellar ataxia and HSP seems artificial, as it reveals a phenotypic continuum linked to specific genes, which can be better understood through the concept of variable expressivity: indeed, little is known about any cis–trans regulatory elements of *GBA2*.

Our MRI findings demonstrate slight variations compared to those reported in the literature (Tables 1 and 5). Specifically, we observed a lower incidence of cerebellar atrophy (0/5), a higher incidence of brainstem atrophy (40% versus 7%), and significant differences concerning WMA (80% versus 15%). These findings highlight the importance of WMA sign in our cohort. However, it is worth noting that our study includes a limited number of cases, and further expansion of the cohort is necessary to enable a more meaningful comparison (Tables 1 and 5).

We have noted a significant reduction in GBA2 activity, as confirmed by tests conducted in our study as well in a few others [20, 23, 26, 27, 29, 47]. Importantly, previous reports and mice studies suggest that this enzyme, despite being relatively understudied, may affect axonal differentiation and branching [69] and locomotor function [20, 24]. Also, GBA2 shows species-specificity [70, 71], especially regarding male reproduction [72, 73], implying that there is more than meets the eye. GBA2 activity test is undoubtedly useful from a diagnostic point of view (Table 1). In our congenital case, it is noteworthy that GBA2 activity was nearly absent, exhibiting a significant reduction compared to proband A, who presented a more typical onset and progression (Table 1).

## Conclusion

In this study, we present a large cohort of Italian SPG46 patients, harbouring a total of seven *GBA2* variants, five of which are novel. The associated phenotype aligns with the overall SPG46 phenotype described in previous reports, showing characteristic features of “typical” ARHSP syndrome and hallmarks like early onset and slow progression of disease, cognitive impairment, scoliosis and cataract. Noteworthy, one case represents the first documented congenital presentation of this condition, and another has the longest disease duration thus far. We identified UGP in two cases of our cohort. It also appears to be relatively frequent in the existing literature. Additionally, movement disorders like tremor and dystonia were observed in several patients. We propose a possible underlying mechanism for UGP and point out the clinical value of this symptom. Also, we highlight tremor and dystonic disorders as clinical specific traits. Unfortunately, there is a high number of papers with unreported data (Tables 3, 4 and 5), limiting informativeness for natural history studies. Brain MRI displayed distinctive SPG46 features, with higher WMA incidence. Dosage of GBA2 activity can be highly beneficial in the diagnostic process, to reduce the time to a confirmatory gene testing. The minor GBA2 activity in our congenital case may suggest a correlation between enzymatic activity and onset, duration, or severity of the disease; but it is hard to speculate on the possible correlations between clinical features and residual enzyme activity since the number of enzyme tests is poor. Biochemical tests can aid in early diagnosis, interdisciplinary management, and assist in potential therapeutic interventions. Future availability of glucosylceramide synthase inhibitors [74, 75] may constitute an efficient and cost-effective genetic testing strategy. It is vital to emphasize the significance of comprehensive observational reports, as they provide valuable information, which enhance our understanding of ultra-rare diseases such as SPG46.

## Data Availability

The authors confirm that the data supporting the findings of this study are available within the article (specifically, Tables 1, 2, 3, 4, 5 and Figures 1, 2A, 2B). More specific clarifications about data are available, upon reasonable request, from the corresponding author (E.C.).
